# Comparative Safety of Robotic-Assisted vs Laparoscopic Cholecystectomy

**DOI:** 10.1001/jamasurg.2023.4389

**Published:** 2023-09-20

**Authors:** Stanley Kalata, Jyothi R. Thumma, Edward C. Norton, Justin B. Dimick, Kyle H. Sheetz

**Affiliations:** 1 Department of Surgery, University of Michigan, Ann Arbor; 2 Center for Healthcare Outcomes and Policy, University of Michigan, Ann Arbor; 3 Department of Health Management and Policy, University of Michigan, Ann Arbor; 4 Department of Economics, University of Michigan, Ann Arbor; 5 Section Editor, *JAMA Surgery*; 6 Department of Surgery, University of California, San Francisco

## Abstract

**Importance:**

Robotic-assisted cholecystectomy is rapidly being adopted into practice, partly based on the belief that it offers specific technical and safety advantages over traditional laparoscopic surgery. Whether robotic-assisted cholecystectomy is safer than laparoscopic cholecystectomy remains unclear.

**Objective:**

To determine the uptake of robotic-assisted cholecystectomy and to analyze its comparative safety vs laparoscopic cholecystectomy.

**Design, Setting, and Participants:**

This retrospective cohort study used Medicare administrative claims data for nonfederal acute care hospitals from January 1, 2010, to December 31, 2019. Participants included 1 026 088 fee-for-service Medicare beneficiaries 66 to 99 years of age who underwent cholecystectomy with continuous Medicare coverage for 3 months before and 12 months after surgery. Data were analyzed August 17, 2022, to June 1, 2023.

**Exposure:**

Surgical technique used to perform cholecystectomy: robotic-assisted vs laparoscopic approaches.

**Main Outcomes and Measures:**

The primary outcome was rate of bile duct injury requiring definitive surgical reconstruction within 1 year after cholecystectomy. Secondary outcomes were composite outcome of bile duct injury requiring less-invasive postoperative surgical or endoscopic biliary interventions, and overall incidence of 30-day complications. Multivariable logistic analysis was performed adjusting for patient factors and clustered within hospital referral regions. An instrumental variable analysis was performed, leveraging regional variation in the adoption of robotic-assisted cholecystectomy within hospital referral regions over time, to account for potential confounding from unmeasured differences between treatment groups.

**Results:**

A total of 1 026 088 patients (mean [SD] age, 72 [12.0] years; 53.3% women) were included in the study. The use of robotic-assisted cholecystectomy increased 37-fold from 211 of 147 341 patients (0.1%) in 2010 to 6507 of 125 211 patients (5.2%) in 2019. Compared with laparoscopic cholecystectomy, robotic-assisted cholecystectomy was associated with a higher rate of bile duct injury necessitating a definitive operative repair within 1 year (0.7% vs 0.2%; relative risk [RR], 3.16 [95% CI, 2.57-3.75]). Robotic-assisted cholecystectomy was also associated with a higher rate of postoperative biliary interventions, such as endoscopic stenting (7.4% vs 6.0%; RR, 1.25 [95% CI, 1.16-1.33]). There was no significant difference in overall 30-day complication rates between the 2 procedures. The instrumental variable analysis, which was designed to account for potential unmeasured differences in treatment groups, also showed that robotic-assisted cholecystectomy was associated with a higher rate of bile duct injury (0.4% vs 0.2%; RR, 1.88 [95% CI, 1.14-2.63]).

**Conclusions and Relevance:**

This cohort study’s finding of significantly higher rates of bile duct injury with robotic-assisted cholecystectomy compared with laparoscopic cholecystectomy suggests that the utility of robotic-assisted cholecystectomy should be reconsidered, given the existence of an already minimally invasive, predictably safe laparoscopic approach.

## Key Points

**Question:**

What are the utilization rates and comparative safety outcomes of robotic-assisted cholecystectomy vs laparoscopic cholecystectomy across the United States?

**Findings:**

This cohort study of claims data for Medicare beneficiaries identified 1 026 088 who underwent a cholecystectomy from 2010 through 2019. Robotic-assisted cholecystectomy rates increased 37-fold, and this procedure was associated with a higher rate of bile duct injury compared with laparoscopic cholecystectomy (0.7% vs 0.2%).

**Meaning:**

In the absence of other advantages over an already minimally invasive procedure, the findings of this study call into question the role of the robotic platform for cholecystectomy.

## Teaser

This cohort study compares the rates and safety outcomes of robotic-assisted cholecystectomy with laparoscopic cholecystectomy in a nationally representative population of older adults.

## Introduction

The US is a global outlier in the adoption of robotic-assisted surgery.^1,2^ While the technology was initially marketed for its ability to overcome technical challenges associated with certain procedures (eg, prostatectomy), it has since expanded into domains with weaker clinical rationale and limited evidence.^3,4^ Recent studies suggesting inferior oncologic outcomes following robotic-assisted surgery for breast cancer^5^ and cervical cancer^6,7^ prompted the US Food and Drug Administration to release a public safety warning. Evidence in general surgery, the field currently experiencing the fastest adoption of robotic-assisted procedures,^1^ suggests that these patterns may expose patients to harm as individual surgeons learn the new surgical approach.^3,8,9^ For example, while cholecystectomy remains one of the most routine operations performed by general surgeons, a recent several-fold increase in the use of robotic-assisted surgical procedures has driven the ongoing debate over the potential safety implications of this change in practice.^10^

Whether robotic-assisted cholecystectomy leads to even safer outcomes than minimally invasive laparoscopic cholecystectomy remains unclear. Some contend that robotic-assisted cholecystectomy may be safer because it offers 3-dimensional visualization, enhanced instrument articulation to allow for more complex maneuvers, novel ways to visualize biliary anatomy,^11^ and potentially increases a surgeon’s ability to perform difficult procedures in a minimally invasive fashion.^12^ Studies^13,14,15^ comparing the safety of these approaches found equivalency, but are limited to single-center case series inclusive of surgeons with the most robotic-assistance experience. Whether those outcomes reflect current surgical practice, especially as robotic-assisted cholecystectomy is adopted by a larger and potentially more novice group of surgeons, represents crucial information for surgeons, referring physicians, and patients.

We performed a study of US Medicare beneficiaries to evaluate the comparative safety of robotic-assisted vs laparoscopic cholecystectomy among a diverse group of patients and surgeons. We leveraged regional variation in the adoption of robotic-assisted cholecystectomy over time as an instrumental variable, accounting for both measured and potentially important but unmeasured sources of bias, to compare outcomes following both procedures.

## Methods

This cohort study was deemed exempt from review by the University of Michigan Institutional Review Board, which also waived the requirement to obtain informed consent because data were deidentified. The study followed the Strengthening the Reporting of Observational Studies in Epidemiology (STROBE) reporting guideline.

### Data Source and Study Population

We used Medicare Provider Analysis and Review claims from January 1, 2010, to December 31, 2019, for nonfederal acute care hospitals. The current analyses were performed from August 17, 2022, to June 1, 2023. We selected patients who underwent 23-hour observation or inpatient admissions for cholecystectomy by using *International Classification of Diseases, Ninth Revision* (*ICD-9*), *International Statistical Classification of Diseases and Related Health Problems, Tenth Revision* (*ICD-10*), and *Current Procedural Technology* codes (eTable 1 in Supplement 1). This technique using *ICD-9* and *ICD-10* codes has been previously used to identify robotic-assisted procedures^16^ and verified within a statewide clinical registry that manually abstracts information on operative approaches.^8^ We included beneficiaries aged 66 to 99 years with continuous Medicare fee-for-service coverage for 3 months before and 12 months after the surgical procedure of interest. We excluded patients in Medicare Advantage and patients with gallbladder, bile duct, and liver cancers (eTable 1 in Supplement 1). We collected information on patient demographic characteristics, and comorbidities were identified using *ICD-9* and *ICD-10* diagnosis codes and defined using the Elixhauser method.^17^ Hospitals were identified and linked to hospital referral regions. Hospital referral regions represent regional health care markets and typically include at least 1 tertiary referral hospital.

### Outcomes

Our primary outcome was bile duct injury requiring surgical repair with hepaticojejunostomy or choledochojejunostomy (surgical repairs in which the biliary system of the liver is connected to the small intestine to restore physiologic drainage) within 1 year following cholecystectomy. These 2 surgical procedures are performed as definitive treatments for severe bile duct injury, which was defined using *ICD-9* and *ICD-10* codes for a bile duct injury combined with codes for either operative repair procedure (eTable 1 in Supplement 1).

Our secondary outcomes were postoperative biliary interventions and the overall incidence of postoperative complications. Biliary interventions reflected a composite outcome of an *ICD-9* or *ICD-10* code for bile duct injury and *ICD-9* or *ICD-10* and *Current Procedural Technology* codes for surgical or endoscopic biliary interventions, which included endoscopic retrograde cholangiopancreatography and common bile duct exploration (eTable 1 in Supplement 1). We chose this composite measure as it may capture more minor bile duct injuries that can be managed without a separate invasive surgery. We also calculated incidence of 30-day overall complications and serious complications (incidence of complications with a hospital length of stay >75th percentile).

### Statistical Analysis

We used univariable statistical tests to compare baseline differences among patients who underwent each operation. We used multivariable logistic regression models to evaluate the association between robotic surgery and postoperative complications. All models included the following covariates: age, race and ethnicity, sex, 29 Elixhauser^17^ comorbidities, primary diagnosis, and year. Race and ethnicity (African American or Black, Asian, Hispanic, or White) were abstracted from Medicare's enrollment database, which is derived from patient self-report and submitted to the Social Security Administration. Race and ethnicity were assessed here to provide additional clarity of the demographics of this patient population. All models accounted for clustering of outcomes within hospital referral regions with robust standard errors.

#### Instrumental Variable Analysis

We performed an instrumental variable analysis to address potential confounding from unmeasured differences among patients who underwent robotic-assisted vs laparoscopic surgery.^18^ The directionality of selection bias in this case was not clear. On one hand, patients undergoing robotic-assisted surgery may be healthier at baseline or have less complex anatomy, as surgeons exploring a new operative approach may wish to learn on more straightforward cases. On the other hand, patients undergoing robotic-assisted surgery may be more complex and surgeons may prefer to make use of the potentially advantageous technologies inherent to the robotic platform. An instrumental variable approach allowed us to address the association of selection bias to explicitly compare outcomes among patients who would be considered candidates for either approach, that is, laparoscopic or robotic-assisted procedures.

Our instrumental variable was the proportional use of robotic-assisted cholecystectomy across the hospital referral region in the year prior to a patient’s operation. Prior studies have similar variables exploiting regional and temporal variation in the adoption of a new operation or operative approach to answer comparative safety or effectiveness questions.^9,19^ This instrumental variable met several prespecified criteria for being considered a suitable instrument. First, it was correlated with the exposure (receipt of robotic-assisted cholecystectomy) but not associated with the outcome (bile duct injury) except through its correlation with the exposure. Second, we evaluated the strength of the instrument’s association with receiving robotic-assisted cholecystectomy by calculating the Kleibergen-Paap Wald *F* statistic (*F* = 2.2 × 10^6^). An *F* statistic higher than 10 is typically considered to be a strong instrument.^20^

We used a 2-stage residual inclusion estimation method for our instrumental variable analysis, which has been shown to lead to more consistent estimates than that of other instrumental variable estimation methods, particularly with nonlinear outcomes.^21^ Our first-stage logistic regression modeled a patient’s odds of undergoing robotic-assisted cholecystectomy, adjusted for the instrumental variable (the prior year’s use of robotic-assisted cholecystectomy for the given hospital referral region) and other patient-level factors (age, sex, race and ethnicity, comorbidities, primary diagnosis, and year). We calculated the raw residuals from this first-stage model and included them as covariates in the second-stage model, which estimated the relative risk of bile duct injury adjusted for patient characteristics in a manner identical to our primary analysis. This instrumental variable analysis was also repeated for our secondary outcomes (biliary interventions, overall complications, and serious complications).

#### Sensitivity Analysis

We performed several sensitivity analyses to provide greater context to our primary findings. We examined any potential association that a hospital’s experience with robotics may have with comparative safety by calculating their annual volume of robotic-assisted cholecystectomy procedures, and then stratified hospitals into quartiles of volume. We also conducted stratified analyses based on clinical urgency (elective vs urgent or emergent) and diagnosis (cholecystitis vs all other diagnoses) as well as patient factors (with vs without obesity). Finally, as our study period spanned both *ICD-9* and *ICD-10* diagnosis codes, we conducted separate analyses for the *ICD-9* and *ICD-10* coding periods.

The statistical analysis was performed using Stata, version 17 (StataCorp LLC). We used a 2-sided approach at the .05 significance level, or 95% CIs excluding 1, for all hypothesis testing.

## Results

### Study Cohort and Patterns in Procedure Approach

A total of 1 026 088 fee-for-service Medicare beneficiaries (mean [SD] age, 72.0 [12.0] years; 53.3% female, 46.7% male; 8.9% African American or Black, 1.8% Asian, 3.7% Hispanic, and 82.2% White) underwent cholecystectomy during the study period. The use of robotic-assisted cholecystectomy increased 37-fold from 211 of 147 341 patients (0.1%) in 2010 to 6507 of 125 211 patients (5.2%) in 2019 (Figure). The use of laparoscopic cholecystectomy increased from 111 149 of 147 341 patients (75.4%) in 2010 to 97 427 of 125 211 patients (77.8%) in 2019. The use of open cholecystectomy decreased from 35 981 of 147 341 patients (24.4%) to 21 277 of 125 211 patients (17.0%) patients over the same period. There were 12 819 of 1 025 520 (1.2%) patients who had a bile duct injury necessitating a definitive operative repair. Rates of bile duct injury decreased over time for the cohort for each surgical technique albeit to differing degrees (eTable 2 in Supplement 1). There were 315 (0.3%) bile duct injuries for patients who underwent laparoscopic cholecystectomy in 2010. This decreased to 138 patients (0.1%) by 2019. There were 14 patients (0.7%) who underwent a robotic cholecystectomy and had a bile duct injury in 2013, and this rate decreased to 29 patients (0.5%) by 2019.

**Figure.**
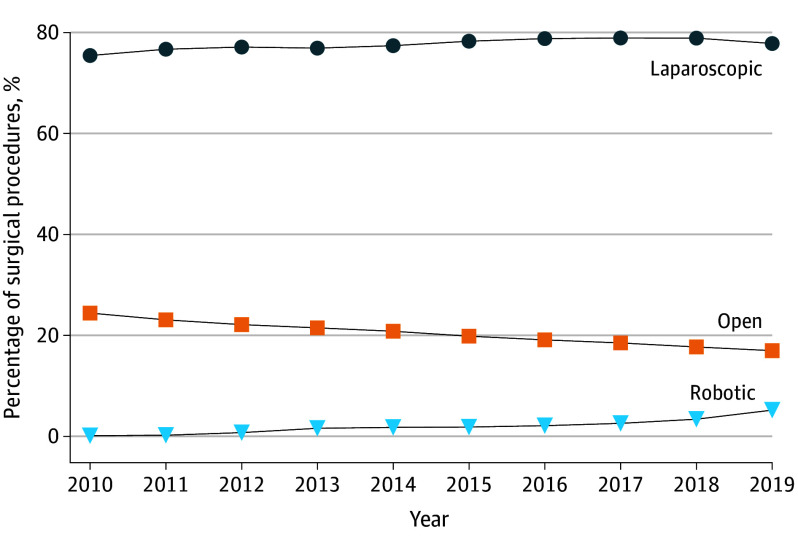
Distribution of Cholecystectomy Techniques Used Among Medicare Beneficiaries by Year From 2010 Through 2019 The number of patients included each year was 147 341 for 2010; 140 100 for 2011; 136 185 for 2012; 131 329 for 2013; 127 36 for 2014; 129 614 for 2015; 129 367 for 2016; 126 494 for 2017; 124 738 for 2018; and 125 211 for 2019.

Of 1 001 004 patients who underwent laparoscopic surgery vs 25 084 who underwent robotic-assisted surgery, those who underwent robotic-assisted surgery were younger (mean [SD] age, 71.2 [11.6] vs 72.0 [12.0] years, *P* < .001) and more likely to be African American or Black (10.4% vs 8.9%, *P* < .001) or Hispanic (4.2% vs 3.7%, *P* < .001) (Table 1). Patients who underwent robotic-assisted surgery also had a higher comorbidity burden, with at least 2 comorbidities (19 840 [79.1%] vs 769 199 [76.8%], *P* < .001). These patient were also more likely to have an elective cholecystectomy (8463 [33.7%] vs 147 220 [14.7%], *P* < .001) and less likely to undergo a cholecystectomy for cholecystitis (19 587 [78.1%] vs 877 529 [87.7%], *P* < .001). Baseline characteristics of the cohort by minimally invasive procedure type is given in Table 1. Most of the patients who underwent an open cholecystectomy were diagnosed as having acute or chronic cholecystitis (rather than other diagnoses, such as gallstone pancreatitis or biliary dyskinesia) (eTable 3 in Supplement 1). Open cholecystectomy was associated with an adjusted rate of 2.9% for bile duct injury and 30.1% for overall complications (eTable 3 in Supplement 1).

**Table 1.** Patient Characteristics for Included Participants From 2010 Through 2019, by Surgical Procedure

Characteristic	Participants, No. (%)		*P* value
	Laparoscopic (n = 1 001 004)	Robotic (n = 25 084)	
Age, mean (SD), y	72.0 (12.0)	71.2 (11.6)	<.001
Sex			
Female	533 392 (53.3)	13 660 (54.5)	<.001
Male	467 612 (46.7)	11 424 (45.5)	
Race and ethnicity			
African American or Black	88 681 (8.9)	2620 (10.4)	<.001
Asian	17 559 (1.8)	486 (1.9)	
Hispanic	36 877 (3.7)	1061 (4.2)	
White	823 135 (82.2)	19 988 (79.7)	
≥2 Elixhauser comorbidities	769 199 (76.8)	19 840 (79.1)	<.001
Hypertension	724 363 (72.4)	18 691 (74.5)	<.001
Fluid or electrolyte disorder	303 768 (30.4)	7807 (31.1)	.008
Diabetes without chronic complications	222 993 (22.3)	5262 (21)	<.001
Chronic obstructive pulmonary disease	195 695 (19.6)	5064 (20.2)	.012
Obesity	177 532 (17.7)	5283 (21.1)	<.001
Hypothyroidism	162 196 (16.2)	4313 (17.2)	<.001
Anemia	151 605 (15.2)	4224 (16.8)	<.001
Kidney failure	150 035 (15)	4031 (16.1)	<.001
Congestive heart failure	122 976 (12.3)	3066 (12.2)	.77
Depression	106 951 (10.7)	2946 (11.7)	<.001
Diabetes with chronic complications	93 657 (9.4)	3317 (13.2)	<.001
Liver disease	77 466 (7.7)	2502 (10)	<.001
Other neurological disorders	76 582 (7.7)	1904 (7.6)	.72
Peripheral vascular disease	67 967 (6.8)	1524 (6.1)	<.001
Valvular disease	66 923 (6.7)	1689 (6.7)	.77
Weight loss	54 790 (5.5)	1567 (6.3)	<.001
Coagulopathy	54 391 (5.4)	1400 (5.6)	.31
Rheumatoid arthritis or collagen vascular disease	33 704 (3.4)	904 (3.6)	.04
Psychoses	33 562 (3.4)	730 (2.9)	<.001
Paralysis	23 317 (2.3)	568 (2.3)	.50
Pulmonary circulation disease	18 315 (1.8)	315 (1.3)	<.001
Solid tumor without metastasis	15 050 (1.5)	505 (2)	<.001
Alcohol use disorder	13 099 (1.3)	390 (1.6)	.001
Metastatic cancer	11 112 (1.1)	526 (2.1)	<.001
Admission type			
Elective	147 220 (14.7)	8463 (33.7)	<.001
Urgent or emergent	853 784 (85.3)	16 621 (66.2)	
Diagnoses			
Cholecystitis	877 529 (87.7)	19 587 (78.1)	<.001
Other diagnoses	123 475 (12.3)	5497 (21.9)	

### Comparative Safety of Laparoscopic vs Robotic-Assisted Cholecystectomy

Robotic-assisted cholecystectomy was associated with higher risk of bile duct injury necessitating a definitive operative repair within 1 year (0.7%) compared with laparoscopic cholecystectomy (0.2%; relative risk [RR], 3.16 [95% CI, 2.57-3.75]). Compared with laparoscopic cholecystectomy, robotic-assisted cholecystectomy was also associated with higher rates of biliary interventions (7.4% vs 6.0%; RR, 1.25 [95% CI, 1.16-1.33]) and serious complications (9.3% vs 8.6%; RR, 1.08 [95% CI, 1.03-1.13]). There was no significant difference between the 2 procedures in 30-day overall complication rates (20.5% vs 20.6%; RR, 1.00 [95% CI, 0.97-1.03]) (Table 2).

**Table 2.** Conventional Multivariable Analysis of Primary and Secondary Outcomes

Adjusted outcome^a^	Type of surgery		Absolute difference, % (95% CI)	Relative risk (95% CI)
	Robotic assisted, % (n = 25 084)	Laparoscopic, % (n = 1 001 004)		
Bile duct injury	0.7	0.2	0.49 (0.36 to 0.62)	3.16 (2.57 to 3.75)
Biliary intervention^b^	7.4	6.0	1.48 (0.97 to 1.98)	1.25 (1.16 to 1.33)
Any complication	20.5	20.6	−0.06 (−0.66 to 0.55)	1.00 (0.97 to 1.03)
Serious complication	9.3	8.6	0.70 (0.28 to 1.13)	1.08 (1.03 to 1.13)

^a^ Outcomes reflected adjusted means, controlling for age, sex, race and ethnicity, 29 Elixhauser comorbidities, diagnosis, and year and clustered within hospital referral regions.

^b^ Postoperative biliary interventions included endoscopic retrograde cholangiopancreatography or common bile duct exploration.

### Instrumental Variable and Sensitivity Analyses

The instrumental variable balanced measured patient characteristics stratified about the median of the instrumental variable (eTable 4 in Supplement 1). In our instrumental variable analysis, robotic-assisted cholecystectomy was still associated with higher risk of bile duct injury compared with laparoscopic cholecystectomy (0.4% vs 0.2%; RR, 1.88 [95% CI, 1.14-2.63]) (Table 3). We observed consistent findings suggesting that robotic-assisted cholecystectomy was associated with higher risk of bile duct injury across multiple sensitivity analyses. For example, robotic-assisted cholecystectomy was associated with higher rates of bile duct injury for elective surgical procedures (1.3% vs 0.5%; RR, 2.64 [95% CI, 2.04-3.25]), urgent or emergent surgical procedures (0.3% vs 0.2%; RR, 1.87 [95% CI, 1.36-2.38]), and for patients who underwent a cholecystectomy for acute or chronic cholecystitis (0.5% vs 0.2%; RR 2.29 [95% CI, 1.79-2.79]) (Table 3). Our instrumental variable analysis of secondary outcomes did not find any statistically significant associations for biliary interventions or complications between robotic-assisted cholecystectomy and laparoscopic cholecystectomy (eTable 5 in Supplement 1).

**Table 3.** Multivariable Sensitivity Analysis of Bile Duct Injury

Adjusted outcome by subgroup^a^	Type of surgery		Absolute difference, % (95% CI)	Relative risk, % (95% CI)
	Robotic assisted, %	Laparoscopic, %		
Instrumental variable analysis^b^	0.4	0.2	0.20 (0.04-0.36)	1.88 (1.14-2.63)
Patients undergoing elective surgery	1.3	0.5	0.81 (0.55-1.08)	2.64 (2.04-3.25)
Patients undergoing urgent or emergent surgery	0.3	0.2	0.16 (0.07-0.25)	1.87 (1.36-2.38)
Cholecystitis	0.5	0.2	0.28 (0.17-0.39)	2.29 (1.79-2.79)
Other diagnoses	1.5	0.3	1.24 (0.84-1.64)	5.14 (3.58-6.69)
Obesity				
With	0.44	0.2	0.25 (0.04-0.47)	2.33 (1.17-3.49)
Without	0.8	0.2	0.55 (0.40-0.69)	3.32 (2.69-3.96)
Robotic-assisted surgery hospital volume				
Highest quartile	0.6	0.2	0.36 (0.21-0.51)	2.46 (1.81-3.11)
Lowest quartile	1.1	0.2	0.87 (0.03-1.71)	4.72 (1.15-8.29)
*ICD-9* (2010-Sep 2015)	0.9	0.3	0.64 (0.40-0.88)	3.37 (2.46-4.28)
*ICD-10* (Oct 2015-2019)	0.5	0.2	0.34 (0.22-0.45)	2.98 (2.29-3.67)

Abbreviations: *ICD-9*, *International Classification of Diseases, Ninth Revision*; *ICD-10*, *International Statistical Classification of Diseases and Related Health Problems, Tenth Revision*.

^a^ Outcomes reflected adjusted means controlling for age, sex, race and ethnicity, 29 Elixhauser comorbidities, diagnosis, and year and clustered within hospital referral regions.

^b^ The relative risk estimates from the instrumental variable analysis represent the local mean treatment effect associated with robotic surgery for patients who would be considered candidates for either surgical approach.

## Discussion

This cohort study of Medicare beneficiaries in the US compared the short-term safety of laparoscopic cholecystectomy, the current standard of care, to robotic-assisted cholecystectomy, a novel and potentially advantageous technology rapidly being adopted into practice. We found that use of robotic-assisted cholecystectomy increased 37-fold between 2010 and 2019. Robotic-assisted cholecystectomy was associated with higher rates of bile duct injury requiring subsequent surgical management and biliary interventions (eg, endoscopic stenting via endoscopic retrograde cholangiopancreatography), which was confirmed in our instrumental variable analysis. Overall complication rates were, on the other hand, similar between laparoscopic and robotic-assisted surgery. Taken together, our data suggest that the utility of robotic-assisted cholecystectomy should be reconsidered, given the availability of an already minimally invasive, predictably safe laparoscopic approach.

The overall rates of bile duct injury in this study are consistent with prior population-based analyses, particularly for patients who underwent inpatient laparoscopic cholecystectomy.^22,23,24^ Single-center retrospective cohort studies^13,14^ comparing laparoscopic and robotic-assisted cholecystectomy have previously suggested comparable rates of postoperative complications, while acknowledging higher total hospital costs attributable to robotics (eg, $8870 for robotic-assisted vs $5771 for laparoscopic approaches^13^). One study^25^ of surgical procedures included in the national inpatient sample found higher overall complication rates for robotic-assisted cholecystectomy compared with laparoscopic cholecystectomy (15.5% vs 11.7%), but this was perhaps attributable to the higher comorbidity burden of patients who underwent robotic-assisted surgery (mean, 2.2 vs 1.9 comorbid conditions).^25,26^ Our work fills 2 key gaps in knowledge around the comparative outcomes of robotic-assisted surgery. First, we specifically provided estimates for rates of technical complications, such as bile duct injury, in a nationally representative sample of patients and surgeons. Second, we leveraged variations in how robotic-assisted surgery was adopted to address issues such as selection bias that may lead to incorrect conclusions about why robotic-assisted cholecystectomy may or may not be safer than laparoscopic cholecystectomy. In other words, we provided a specific mechanism by which robotic-assisted cholecystectomy was less safe despite the purported benefits inherent to the technology.

Our results reinforce an important longitudinal pattern that globally affects the quality of care for numerous patients undergoing cholecystectomy each year: the rates of open cholecystectomy continue to decline.^27^ When laparoscopic cholecystectomy was introduced approximately 30 years ago as a minimally invasive alternative to open surgery, it was appropriately criticized for higher rates of technical complications, such as common bile duct injury.^28,29^ This complication results in a cascade of endoscopic or highly morbid surgical interventions (4.0%-6.5% 30-day mortality)^30,31^ that decrease long-term survival.^30,32^ Increasing familiarity with laparoscopy along with deliberate continuing education programs, such as the SAGES Safe Cholecystectomy Program,^33,34,35^ have rendered laparoscopic cholecystectomy the standard of care, with lower complication rates and faster recovery compared with open surgery.^22,24,27^ The present study further supports the success of those initiatives, as the rate of bile duct injury for laparoscopic cholecystectomy in 2010 was 0.3% and dropped to 0.1% by 2019. On the other hand, robotic-assisted cholecystectomy had a 0.4% bile duct injury rate by 2019 for the 5.2% of Medicare beneficiaries who underwent cholecystectomy that year. Unfortunately, the clinical justification to devote time and resources to expose patients to increased risk as surgeons progress along the procedural learning curve from laparoscopic to robot-assisted cholecystectomy remains theoretical.

The extent to which the pattern of moving away from open surgery is further driven by the adoption of robotic-assisted surgery, with the potential to allow more difficult operations to be performed in a minimally invasive fashion, is unclear. Our findings do not suggest that the growth of robotic-assisted cholecystectomy is being driven by these more complex cases that would have otherwise been performed by an open approach. First, we found that robotic-assisted cholecystectomy was more likely to be elective and performed for biliary colic or gallstone pancreatitis rather than for acute or chronic cholecystitis. This finding may suggest that surgeons are using this technique for lower-risk cholecystectomy procedures while they are learning the technique. Furthermore, if robotic-assisted cholecystectomy was being performed on candidates who were not laparoscopic cholecystectomy candidates, the rates of bile duct injury in the open group would increase over time as only the highest-risk patients would remain. Instead, rates of bile duct injury continued to decrease for both laparoscopic and open cholecystectomy approaches. Finally, overall complication rates between robotic-assisted and laparoscopic surgical procedures were similar. As a result, it is plausible that at least some of the robotic-assisted cholecystectomy procedures are replacing laparoscopic cholecystectomy. This has been observed in other clinical domains. For example, the adoption of robotic-assisted colectomy was associated with a 2:1 replacement of laparoscopic surgical procedures (already minimally invasive) over open ones.^9^ Beyond clinical justifications, industry marketing and patient preferences (eg, assumptions that new technology renders superior outcomes) have also been implicated in the expansion of robotic-assisted surgery across a range of clinical specialties.^36,37^

Surgeons remain optimistic that the robotic platform will increase patients’ access to minimally invasive operations even if there are some practical trade-offs to consider. For example, there is a large body of evidence suggesting that robotic-assisted surgery is more expensive than laparoscopy, has longer operative times, and requires more specialized operating room staff.^13,14,24,25^ Even though some randomized controlled trials comparing robotic-assisted and laparoscopic surgery for rectal cancer resection or inguinal hernia repair did not demonstrate differences in outcomes, many studies contend that there are benefits from enabling surgeons to perform these technically complex operations in a minimally invasive fashion.^38,39^ In other words, the technical challenges of performing those operations laparoscopically may drive surgeons to pursue mastery of the potentially more accommodating robotic-assisted alternative.^3^

For cholecystectomy, however, the traditional laparoscopic approach is a core competency for practicing surgeons and general surgical trainees. In the absence of a clear safety advantage, it is not clear what problem the robotic technology is addressing. Because cholecystectomy is such a familiar operation, there may be other reasons behind the adoption of robotic-assisted cholecystectomy. For example, surgeons may leverage their familiarity with cholecystectomy to practice their robotic-assisted surgical skills.^15^ But this justification and others related to expanding clinical applications for robotic-assisted surgery may need to be more carefully balanced against any appreciable disadvantages related to patient safety.

### Limitations

This study has limitations. Because this was a retrospective study using administrative claims data, our results relied on accurate coding of diagnoses, comorbidities, and postoperative complications. Limitations associated with unmeasured variables, such as sociodemographic factors, comorbidities, and surgical complexity, may also have biased our results. We were unable to capture procedural factors that have been shown to play a crucial role in the development of bile duct injuries, such as the severity and chronicity of gallbladder inflammation. Furthermore, we were unable to identify intraoperative conversions in the data, which limits our ability to comment on the outcomes associated with converting from one procedure to another. While rare, intraoperative conversions have been associated with significantly higher rates of bile duct injury in a previous study.^23^ In addition, we were unable to quantify using claims data whether the robotic platform was increasing the number of patients who could receive a minimally invasive operation or whether the robotic platform was simply replacing the laparoscopic approach. However, we attempted to control for these issues in multiple ways. First, we used diagnoses and outcomes reliably coded in claims data. Second, we specifically focused on a primary outcome that was previously used in similar studies^22,32^ and is less subject to coding discrepancies. Finally, we performed an instrumental variable analysis to control for unmeasured factors that may bias our comparisons between both procedure approaches. This study also focused on the Medicare population that underwent inpatient or observation status procedures, which may limit the generalizability of our findings. However, cholecystectomy remains one of the most common general surgical operations performed in individuals of all ages, and we would not expect the results to differ purely on age (ie, cases are managed similarly regardless of patient age).

## Conclusions

This cohort study found that, among Medicare beneficiaries, the use of robotic-assisted cholecystectomy increased from 2010 through 2019 and was associated with higher rates of bile duct injury compared with laparoscopic cholecystectomy. In the absence of other advantages over an already minimally invasive procedure, these data call into question the practice of robotic-assisted cholecystectomy.

## Notes

Supplement 1. **eTable 1.** *CPT*, *ICD-9*, and* ICD-10 *Codes Used to Define Covariates and Outcomes **eTable 2.** Unadjusted Rates of Bile Duct Injury Stratified by Surgical Technique Over Time **eTable 3.** Patient Demographics and Adjusted Outcomes for Patients Undergoing Open Cholecystectomy, 2010-2019 **eTable 4.** Demographics Stratified by the Median of the Instrumental Variable (Prior Year Robotic Utilization Rate), 2011-2019 **eTable 5.** Instrumental Variable Analysis of Secondary Outcomes

Supplement 2.
Data Sharing Statement
