# Supplementary material for: Comparative Safety of Robotic-Assisted vs Laparoscopic Cholecystectomy
Source: JAMA Surg. 2023 Sep 20;158(12):1303–10. doi: 10.1001/jamasurg.2023.4389 (PMC10512167; doi:10.1001/jamasurg.2023.4389)
Supplement: Supplement 1. — eTable 1. CPT, ICD-9, and ICD-10 Codes Used to Define Covariates and Outcomes eTable 2. Unadjusted Rates of Bile Duct Injury Stratified by Surgical Technique Over Time eTable 3. Patient Demographics and Adjusted Outcomes for Patients Undergoing Open Cholecystectomy, 2010-2019 eTable 4. Demographics Stratified by the Median of the Instrumental Variable (Prior Year Robotic Utilization Rate), 2011-2019 eTable 5. Instrumental Variable Analysis of Secondary Outcomes [file jamasurg-e234389-s001.pdf]

Supplementary Online Content

Kalata S, Thumma JR, Norton EC, Dimick JB, Sheetz KH. Comparative safety of robotic-assisted vs laparoscopic cholecystectomy. *JAMA Surg*. Published online September 20, 2023. doi:10.1001/jamasurg.2023.4389

eTable 1. *CPT*, *ICD-9*, and *ICD-10* Codes Used to Define Covariates and Outcomes

eTable 2. Unadjusted Rates of Bile Duct Injury Stratified by Surgical Technique Over Time

eTable 3. Patient Demographics and Adjusted Outcomes for Patients Undergoing Open Cholecystectomy, 2010-2019

eTable 4. Demographics Stratified by the Median of the Instrumental Variable (Prior Year Robotic Utilization Rate), 2011-2019

eTable 5. Instrumental Variable Analysis of Secondary Outcomes

This supplementary material has been provided by the authors to give readers additional information about their work.

© 2023 Kalata S et al. *JAMA Surgery.*

eTable 1. *CPT, ICD-9,* and *ICD-10* Codes Used to Define Covariates and Outcomes

**Variable**	**CPT/ICD-9/ICD-10 codes**
Cholecystectomy	CPT:49310, 56340, 56342, 47562, 47564, 47600, 47610, 47612, 47620
Cholecystectomy with IOC	CPT:49311, 56341, 47563, 47605
Hepatobiliary, pancreatic, or duodenal malignancies	ICD-9: 152*, 155*, 156*, 157*, 158*, 159*
Biliary colic and/or biliary dyskinesia	ICD-9: 574.2*, 789.00, 789.01, 574.50, 574.70, 575.8, 5752, 5753, 5754, 5755, 5756, 5758, 5759, 576, 5761, 5762, 5763, 5764, 5765, 5768, 5769,<br>ICD-10: K82, K820, K821, K822, K823, K824, K828, K829, K83, K830, K831, K832, K833, K834, K835, K838, K839
Common duct stones/Gallstone pancreatitis*	ICD-9: 574.3*-574.9*/ 577.0
Cholecystitis	ICD-9: 5740, 57400, 57401, 57410, 57411, 5743, 57430, 57431, 57440, 57441, 5746, 57460, 57461, 57470, 57471, 5748, 57480, 57481, 575, 5750, 5751, 57511, 57512, 57510<br>ICD-10: K81.0, K81.1, K81.2, K81.9, K800, K8000, K8001, K801, K8010, K8011, K8012, K8013, K8018, K8019, K804, K8040, K8041, K8042, K8043, K8044, K8045, K8046, K8047, K806, K8060, K8061, K8062, K8063, K8064, K8065, K8066, K8067, K810, K811, K812, K819
Cholelithiasis	ICD-9 - 5742, 57420, 57421, 57441<br>ICD-10 - K80, K82, K83, K802, K8020, K8021, K808, K8080, K8081
Gallstone Pancreatitis	ICD-9: 5742, 57420, 57421, 57441, 5770<br>ICD-10: K85.10, K85.11, K85.12
Gallbladder/HPB Cancer	ICD-9: 156.0, 156.1, 156.2, 156.8, 156.9,155.1, 157<br>ICD-10: C22.1 C22.8 C22.9 C23 C24 C24.0 C24.1 C24.9 C25.0
Open cholecystectomy	CPT:47600, 47605, 47610, 47612, 47620
Laparoscopic cholecystectomy	CPT:49310, 49311, 47562, 47563, 47564, 56340, 56341, 56342
Robotic identifier codes	ICD-9: 17.41-17.44, 17.49<br>ICD-10: 8E0W0CZ, 8E0W3CZ, 8E0W4CZ, 8E0W7CZ, 8E0W8CZ
Bile Duct Injury	ICD-9: 576.3, 576.4, 576.0, 868.02, 995, 997.4, 997.49, 998.13, 998.2, 998.51, 998.59, 998.89<br>ICD-10: S36.13, S36. 13XA, S36. 13XD, S36. 13XS, K83.2
Choledochojejunostomy/hepaticojejunostomy	ICD-9: 51.94, 51.36, 51.37, 51.39, 51.79, 51.72<br>ICD10:0F15 0F16 0F17 0F19<br>CPT:47701, 47720, 47721, 47740, 47760, 47765, 47780 , 47785,
Fistula of the bile duct	ICD-9: 576.4
Endoscopic retrograde cholangiopancreatography (ERCP)	ICD-9: 51.10, 51.11, 51.84-51.88, 52.13, 51.99, 52.93, 52.94<br>ICD10: 0FJB* 0FHB* 0FQ7* 0FQ9* 0FR7* 0FR9*<br>CPT: 43260-43262, 43264-43269, 43271 , 43274, 43275, 43276
Common duct exploration	ICD-9: 51.41, 51.43, 51.49, 51.51; CPT: 47564, 47610<br>CPT: 47564, 47610

Gallstone pancreatitis included any record with any discharge diagnosis code for gallstones (574* or 575* with any extension) and a diagnosis of acute pancreatitis (577.0)

© 2023 Kalata S et al. *JAMA Surgery.*

eTable 2. Unadjusted Rates of Bile Duct Injury Stratified by Surgical Technique Over Time

Year	Open (%)	Laparoscopic (%)	Robotic (%)
2010	1,427 (4.0%)	315 (0.28%)	**
2011	1,382 (4.0%)	272 (0.26%)	**
2012	1,252 (4.2%)	274 (0.26%)	**
2013	1,178 (4.2%)	261 (0.26%)	14 (0.67%)
2014	1,141 (4.3%)	283 (0.29%)	19 (0.83%)
2015	1,099 (4.3%)	241 (0.24%)	20 (0.83%)
2016	763 (3.1%)	187 (0.18%)	16 (0.59%)
2017	728 (3.1%)	174 (0.17%)	21 (0.64%)
2018	705 (3.2%)	183 (0.19%)	25 (0.59%)
2019	649 (3.1%)	138 (0.14%)	29 (0.45%)
**Total**	10,324 (3.8%)	2,328(0.23%)	167(0.67%)

**Medicare data are not presented in these cells due to small sample size

© 2023 Kalata S et al. *JAMA Surgery.*

eTable 3. Patient Demographics and Adjusted Outcomes for Patients Undergoing Open Cholecystectomy, 2010-2019

**Characteristics**	**N=264,681**
Age, mean (SD)	72.1 (10.9)
Female (%)	121,182 (45.6%)
Race/Ethnicity	
White (%)	216,526 (81.8%)
Black (%)	27,544 (10.4%)
Hispanic (%)	6,738 (2.6%)
Asian (%)	4,679 (1.8%)
2+ Elixhauser Comorbidities (%)	209,448 (79.1%)
Admission type	
Elective (%)	109,760 (41.5%)
Urgent/Emergent (%)	154,921 (58.5%)
Diagnoses	
Cholecystitis (%)	188,516 (71.2%)
Other diagnoses (%)	76,165 (28.8%)
**Adjusted Outcomes**	
Bile Duct Injury	2.9%
Biliary Intervention*	14.25%
Any Complications	30.08%
Serious Complications	19.46%

Outcomes reflected adjusted averages controlling for age, gender, race, 29 Elixhauser comorbidities, diagnosis, year and clustered within hospital referral regions. This model included patients who underwent open cholecystectomy, laparoscopic cholecystectomy, and robotic-assisted cholecystectomy.
*Postoperative biliary interventions included endoscopic retrograde cholangiopancreatography or common bile duct exploration

© 2023 Kalata S et al. *JAMA Surgery.*

eTable 4. Demographics Stratified by the Median of the Instrumental Variable (Prior Year Robotic Utilization Rate), 2011-2019

Characteristics	Below Median n=458,509	Above Median n=458,148	Standardized Difference	p-value
Age, mean (SD)	71.98 (12.1)	72.05 (11.8)	0.006	0.001
Female (%)	244,559 (53.3%)	241,673 (52.7%)	0.012	<.0001
Race/Ethnicity				
White (%)	385,763 (84.1%)	365,565 (79.8%)	0.113	<.0001
Black (%)	38,102 (8.3%)	43,547 (9.5%)	0.042	<.0001
Hispanic (%)	13,581 (3.0%)	20,783 (4.5%)	0.083	<.0001
Asian (%)	6,384 (1.4%)	10,146 (2.2%)	0.062	<.0001
2+ Elixhauser Comorbidities (%)	352,365 (76.9%)	363,015 (79.2%)	0.058	<.0001
Hypertension (%)	333,763 (72.8%)	343,514 (75.0%)	0.050	<.0001
Fluid/electrolyte disorders (%)	138,191 (30.1%)	149,431 (32.6%)	0.053	<.0001
Diabetes without chronic complications (%)	109,457 (23.9%)	95,131 (20.8%)	0.075	<.0001
COPD (%)	93,202 (20.3%)	90,021 (19.6%)	0.017	<.0001
Obesity (%)	81,883 (17.9%)	91,499 (20.0%)	0.054	<.0001
Hypothyroidism (%)	76,431 (16.7%)	77,896 (17.0%)	0.009	<.0001
Anemias (%)	72,058 (15.7%)	73,899 (16.1%)	0.011	<.0001
Renal failure (%)	68,944 (15.0%)	74,668 (16.3%)	0.035	<.0001
Congestive heart failure (%)	56,169 (12.3%)	59,005 (12.9%)	0.019	<.0001
Depression (%)	51,325 (11.2%)	51,645 (11.3%)	0.002	0.22
Diabetes with chronic complications (%)	36,146 (7.9%)	57,252 (12.5%)	0.153	<.0001
Liver disease (%)	32,458 (7.1%)	42,670 (9.3%)	0.082	<.0001
Other neurological disorders (%)	35,599 (7.8%)	36,826 (8.0%)	0.010	<.0001
Peripheral vascular disease (%)	32,573 (7.1%)	32,309 (7.1%)	0.002	0.428
Valvular disease (%)	32,283 (7.0%)	32,091 (7.0%)	0.001	0.744
Weight loss (%)	25,481 (5.6%)	25,916 (5.7%)	0.004	0.017
Coagulopathy (%)	25,065 (5.5%)	26,994 (5.9%)	0.018	<.0001
Rheumatoid arthritis/collagen vascular (%)	16,035 (3.5%)	15,886 (3.5%)	0.002	0.515
Psychoses (%)	15,869 (3.5%)	15,191 (3.3%)	0.008	<.0001
Paralysis (%)	10,131 (2.2%)	11,533 (2.5%)	0.020	<.0001
Pulmonary circulation disease (%)	10,316 (2.2%)	6,341 (1.4%)	0.065	<.0001
Solid tumor without metastasis (%)	6,850 (1.5%)	7,330 (1.6%)	0.009	<.0001
Alcohol abuse (%)	4,430 (1.0%)	7,758 (1.7%)	0.064	<.0001
Metastatic cancer (%)	5,081 (1.1%)	5,317 (1.2%)	0.005	0.024

SD=Standard Deviation
COPD=Chronic Obstructive Pulmonary Disease

© 2023 Kalata S et al. *JAMA Surgery.*

eTable 5. Instrumental Variable Analysis of Secondary Outcomes

Adjusted Outcomes	Robotic	Laparoscopic	Absolute Difference (95% CI)	Relative Risk (95% CI)	p***
Biliary Intervention**	5.78%	5.76%	0.02% (-0.8, 0.84)	1.00 (0.86, 1.15)	<0.001
Any Complications	19.84%	21.06%	-1.22% (-2.48, 0.03)	0.94 (0.88, 1.00)	0.023
Serious Complications	9.58%	8.64%	0.95% (-0.02, 1.91)	1.11 (1.00, 1.22)	0.585

The relative risk estimates from the instrumental variable analysis represents the local mean treatment effect of robotic surgery in patients who would be considered candidates for either surgical approach. Outcomes reflected adjusted averages controlling for residuals from first stage of instrumental variable analysis, age, gender, race, 29 Elixhauser comorbidities, diagnosis, year and clustered within hospital referral regions

**Postoperative biliary interventions included ERCP or bile duct exploration

***Value represents significance of Durbin-Wu-Hausman test performed on coefficient of residual from 1st stage of 2-stage residual inclusion estimation method. As this is a direct test of the endogeneity of robotic test, a significant value means that IV estimation is preferred to convention logistic regression.

© 2023 Kalata S et al. *JAMA Surgery.*
