# Supplementary material for: Comparative Safety of Robotic-Assisted vs Laparoscopic Cholecystectomy
Source: JAMA Surg. 2023 Sep 20;158(12):1303–10. doi: 10.1001/jamasurg.2023.4389 (PMC10512167; doi:10.1001/jamasurg.2023.4389)
Supplement: Supplement 2. — Data Sharing Statement [file jamasurg-e234389-s002.pdf]

Data Sharing Statement

Kalata. Comparative Safety of Robotic-Assisted vs Laparoscopic Cholecystectomy. *JAMA Surg*. Published September 20, 2023. doi:10.1001/jamasurg.2023.4389

Data
Data available: No

Additional Information
Explanation for why data not available: We utilized Medicare claims data and are unable to share the data based on our data usage agreement.
